# Amplification Failure of the Amelogenin X Gene Caused by a Rare Mutation in the Primer-Binding Region

**DOI:** 10.3390/genes14111986

**Published:** 2023-10-24

**Authors:** Miwha Chang, Jong Keun Jung, Ji Hwan Park, Ju Yeon Jung, Won-Hae Lee, Joo-Young Kim

**Affiliations:** 1 Forensic DNA Division, National Forensic Service, Wonju-si 26460, Republic of Korea; miwha0106@gmail.com (M.C.); jhpark0613@korea.kr (J.H.P.); 2 Division of Laboratory Diagnosis Management, Korea Disease Control and Prevention Agency, Cheongju-si 28159, Republic of Korea; 3 National Forensic Service, Busan Institute, Yangsan-si 50612, Republic of Korea; jjkcfmo@korea.kr; 4 Jeju Branch, National Forensic Service, Jeju-si 63309, Republic of Korea; jjy7@korea.kr; 5 National Forensic Service, Seoul Institute, Seoul 08036, Republic of Korea; onesun@korea.kr

**Keywords:** amelogenin, AMELX allelic dropout, STR genotyping, point mutation study, gender identification

## Abstract

The study of gender markers is essential in forensic genetic analysis. Mutations in the X or Y homologs of the amelogenin gene can be misleading, resulting in serious mistakes in forensic genetic analysis. We recently discovered two male cases of the X homolog of the amelogenin (AMELX) allelic dropout while analyzing short tandem repeat genotypes obtained from crime scene evidence. Subsequently, we evaluated the molecular characteristics of AMELX allelic dropout in this study. We used two previously reported amelogenin primers to verify a half level of amelogenin gene amplification intensity in the two male cases, which we confirmed was caused by AMELX allelic dropout. We then characterized the point mutation using Sanger sequencing and designed mutation-specific primers that could overcome AMELX allelic dropout. Short tandem repeat genotyping analysis confirmed that the AMELX allelic dropout was recovered by the mutation-specific primer designed specifically for this case. The sequencing of the AMELX allele revealed a single-point variant from A→G at base position 7 downstream from the 3′ end in the amelogenin forward primer-binding region. This point mutation was identically found in two different male cases, resulting in AMELX allelic dropout. To our knowledge, these mutations and the X homolog amplification failure of amelogenin have not been reported in the Korean population. Our study provides a reliable approach to AMELX allelic dropout due to rare case mutations and could enable the better interpretation of gender markers for forensic samples.

## 1. Introduction

The amelogenin gene, which codes for a protein of dental enamel, is located in the sex-specific region of human X (AMELX) and Y (AMELY) chromosomes. These two chromosome homologs differ slightly in size and sequence, allowing the use of the AMEL locus as a sex-typing marker in short tandem repeat (STR) multiplex kits [1,2,3]. The most widely used application of STR analysis containing the AMEL locus is in forensic fields, such as DNA databases, crime scene casework, and missing person familial identification. Recently, DNA typing applications have been widely used in the field of diagnostic medicine, including pathology. Specifically, it is used in prenatal diagnoses to evaluate the origin of tumors inadvertently transmitted by solid organ transplantation and the translocation monitoring of patients who have received allogeneic bone marrow transplants [4,5]. The most commonly used amelogenin primer set flanks a 6 bp deletion within intron 1 of AMELX, producing fragments of 106 bp and 112 bp for the X and Y chromosomes, respectively [6,7,8,9,10]. Both X- and Y-chromosome homologs can be amplified in a single reaction, and the amplification of the amelogenin gene has the advantage of an internal positive control since the X chromosome sequence should always be present [3,11]. Several studies have reported mutations, such as null alleles at the AMELX and AMELY loci [3,6,12,13,14,15,16]. Most Y-specific amplification failures of the amelogenin marker are due to large deletions spanning this locus, whereas X-specific dropout is less common and is usually determined by point mutations at the primer-binding sites. Contrary to AMELY amplification failure, AMELX allelic dropout does not lead to incorrect sex typing because the Y-specific amplification of amelogenin is analyzed in males. However, the absence of the AMELX product can cause a misinterpretation of gender in individuals involved in DNA mixtures [2]. This issue could cause a major problem in solving cases through DNA identification because the suspect and victim’s biological samples are often mixed in crime scene evidence. In this study, we report two male cases with the same mutations in the internal region of the amelogenin forward primer, resulting in a failure to amplify the X homolog of amelogenin. We suggest that a systematic research approach, such as this case study, could help understand AMELX allelic dropout in crime scene samples.

## 2. Materials and Methods

### 2.1. Case Samples

The two AMELX allelic dropout cases described here were analyzed from unidentified DNA samples (confirmed as Koreans through Y-STR and mtDNA analysis) obtained from crime scene evidence received by the National Forensic Service (NFS) over the past three years, which were anonymized to preserve the privacy of the subjects in accordance with the NFS Institutional Review Board requirements (906-220421-BR-007-02).

### 2.2. Isolation and STR Genotyping of Genomic DNA

Genomic DNA from each sample was extracted using the QIAamp^®^ DNA Micro Kit (QIAGEN, Hilden, Germany) according to the manufacturer’s protocol, and autosomal STR was amplified using both the GlobalFiler^TM^ PCR Amplification Kit (Applied Biosystems, Foster City, CA, USA) and the PowerPlex^®^Fusion System (Promega, WI, USA) was used according to the manufacturer’s instructions on a 9700 GeneAmp PCR system (Applied Biosystems). In the experiment using the GlobalFiler^TM^ PCR, 1 µg of case sample DNA, 2.5 µL of the primer set, and 7.5 µL of the master mix was mixed with sterilized, distilled water adjusted to a total reaction volume of 25 µL. The PCR conditions were as follows: initial denaturation at 95 °C for 1 min; 29 cycles of denaturation at 94 °C for 10 s, annealing and extension at 59 °C for 90 s; followed by final extension at 60 °C for 10 min. For PowerPlex^®^Fusion PCR, 1 µg of case sample DNA, 5 µL of the primer pair, and 5 µL of the master mix was mixed with sterilized, distilled water and adjusted to a total reaction volume of 25 µL. The PCR conditions were as follows: initial denaturation at 96 °C for 1 min; 30 cycles of denaturation at 94 °C for 10 s; annealing at 64 °C for 1 min, and extension at 72 °C for 30 s; followed by final extension at 60 °C for 10 min. PCR amplification products were subjected to capillary electrophoresis using a 3500×L genetic analyzer (Applied Biosystems). The 20 µL reaction mixture containing 1 µL of the amplified PCR product, 0.5 µL of the size standard, and 18.5 µL of deionized Hi-Di formamide was denatured at 95 °C for 3 min and chilled for 3 min. Data were analyzed using GeneMapper ID-X v1.4 software (Applied Biosystems).

### 2.3. PCR Amplification of Amelogenin Locus in Genomic DNA

**Figure 1:**
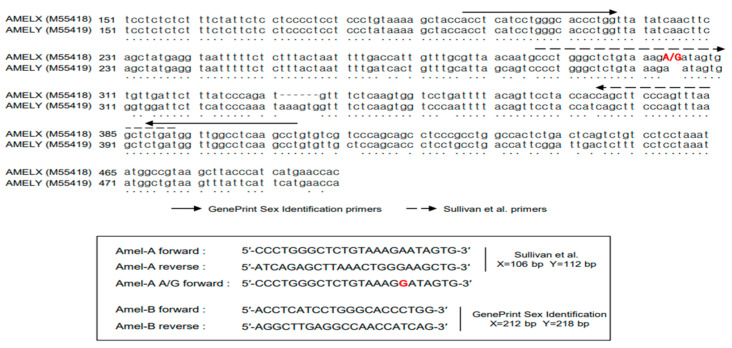
Positions and sequences of oligonucleotide primers used for amelogenin gene amplification (part of GeneBank No. M55418 and M55419 corresponding to the X and Y homolog, respectively, of the amelogenin locus). Amel-A primers generate an X-chromosome-specific product (106 bp) and a Y-chromosome-specific product (112 bp). These primers were used with PowerPlex^®^Fusion, but GlobalFiler™ did not disclose information on amelogenin primers. The Amel-A A/G forward primer substituted the G base for the A base located at the 7th base position from the 3′ end. The point mutation is indicated in red. Amel-B primers generated an X-chromosome-specific product (212 bp) and a Y-chromosome-specific product (218 bp). The nucleotide sequences of primers used in this study are outlined by the rectangle.

For the amelogenin locus amplification of the case samples, Promega GenePrint Sex Identification primers (212/218 bp PCR products) and Sullivan et al. primers (106/112 bp PCR products) [3,7,11] were used (Figure 1). Briefly, 2.5 µL of a 10× Gold ST*R Buffer, 0.4 µmol/L of the amelogenin primer set, 2 units of AmpliTaq Gold^TM^ DNA Polymerase, 1 ng of case sample DNA, and sterilized, distilled water were adjusted to a total reaction volume of 25 µL. The PCR conditions were as follows: initial denaturation at 95 °C for 11 min, 30 cycles of denaturation at 94 °C for 30 s, annealing at 64 °C for 30 s, extension at 72 °C for 45 s, and final extension at 72 °C for 10 min. The amplified PCR product was separated on 4% agarose gel and visualized using an Image Analyzer (UVP GelSolo, Analytik Jena, Jena, Germany). The intensity of the amplified PCR products was measured using ImageJ software v1.54d (open-source program, https://imagej.nih.gov) (accessed on 24 January 2023).

### 2.4. Quantification of Amelogenin Locus in Genomic DNA

For the amelogenin locus quantification of case samples, quantitative real-time PCR was performed using the iQ^TM^ SYBR^®^ Green Supermix kit (Bio-Rad, Hercules, CA, USA) according to the manufacturer’s instructions on a C1000 Touch Thermal Cycler (Bio-Rad). Briefly, 10 µL of a 2× iQ^TM^ SYBR^®^ Green Supermix, 0.4 µmol/L of the amelogenin primer set, 1 ng of case sample DNA, and sterilized distilled water were adjusted to a total reaction volume of 20 µL. The reaction conditions were as follows: 95 °C for 3 min, followed by 40 cycles of 94 °C for 15 s and 64 °C for 30 s. Fluorescence readings were taken during the anneal/extension step (64 °C incubation). Following threshold-dependent cycling, the melting curve reaction was performed from 60 to 95 °C at either 0.1 or 0.4 °C/s melt rates with a smooth curve setting at an average of 1 point. Melting peaks were visualized by plotting the absolute value of the 1st derivative against the temperature. The melting temperature (Tm) was defined as the peak of the curve, and if the highest point was a plateau, the mid-point was identified as the Tm [17].

### 2.5. Sequencing Analysis of Amelogenin Locus

Amel-B primers were purchased from Macrogen (Seoul, Republic of Korea). These primers produced a 212/218 bp fragment for the X/Y chromosome, respectively, as verified by the GenePrint^TM^ Sex Determination System [11]. Amel-B primer information was confirmed by the Promega Corporation. These primers were not fluorescently labeled and were used for the amelogenin locus sequencing of the case samples. Each 25 µL of the PCR reaction contained 2.5 µL of a 10X Gold ST*R Buffer, 5 units of AmpliTaq Gold DNA Polymerase, 10 ng of case sample DNA, and final primer concentrations of 0.4 µmol/L Amel-B forward and reverse primers. The PCR was performed as previously described. PCR products from the case samples were ligated into the pCR4-TOPO TA vector and transformed into a One Shot TOP10 competent *E. coli* provided in the TOPO^TM^ TA cloning^TM^ kit (Invitrogen, Carlsbad, CA, USA) according to the manufacturer’s instructions. Because both X- and Y-chromosome homologs can be generated in a single PCR reaction, individual colonies were picked up to confirm the point mutation of the AMELX locus. Individual colonies (10 colonies from each case sample) were picked and placed individually. PCR was then performed directly on each transformed colony with 25 µL of the PCR master mix. Thermocycling was performed as previously described. The amplification products were purified by adding 12 µL of the product to 5 µL of ExoSAP-IT^®^ (Applied Biosystems), which was reacted for 20 min at 37 °C, followed by 20 min at 80 °C. Purified PCR products were subjected to DNA sequencing using the BigDye^TM^ Terminator v1.1 Cycle Sequencing Kit (Applied Biosystems) on a 3500×L genetic analyzer (Applied Biosystems). 

### 2.6. Allele-Specific Genotyping

Amel-A primers (Macrogen) amplified the X-specific product (106 bp) and Y-specific product (112 bp), as verified by Sullivan et al. [7]. The Amel-A forward and Amel-A A/G forward primers were labeled with 5′ FAM (6-carboxyfluorescein). Each 25 µL PCR reaction contained 2.5 µL of a 10× Gold ST*R Buffer, 2 units of AmpliTaq Gold^TM^ DNA Polymerase, 1 ng of case sample DNA, and final primer concentrations of 0.4 µmol/L 5′ for the FAM-labeled Amel-A or Amel-A A/G forward primer and Amel-A reverse primer. Thermocycling was performed as previously described. PCR amplification products were subjected to capillary electrophoresis using a 3500×L genetic analyzer (Applied Biosystems), and export data were analyzed using GeneMapper ID-X v1.4 software (Applied Biosystems).

## 3. Results

### 3.1. Identification of the AMELX Allelic Dropout in the Forensic Samples

**Figure 2:**
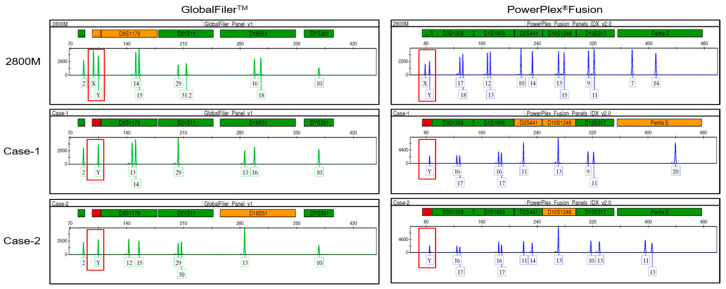
Part of the STR genotyping of AMELX allelic dropout case samples was analyzed using the following two STR genotyping kits: GlobalFiler™ and PowerPlex^®^Fusion. For each kit, we report the output for the AMEL locus (red frames) and the other loci labeled with the same dye. Compared with the positive control DNA (2800 M), AMELX allelic dropout was observed in 2 male cases (Case-1 and Case-2) obtained from crime scene evidence.

A failure to detect diagnostic targets for sex chromosomes in crime scene samples could increase the risk of errors in gender interpretation. However, alternative identification methods have not been sufficiently studied and still remain an important challenge in the field of forensic diagnosis and applications. In this study, we recently discovered two male cases of AMELX allelic dropout when analyzing STR genotypes obtained from crime scene evidence. Figure 2 shows that no AMELX allelic amplification was observed in either case (Case-1 and Case-2) compared to the positive control DNA (2800 M), even though the analysis was performed using two different STR genotyping kits, GlobalFiler™ and PowerPlex^®^Fusion. Based on these results, we attempted to study the molecular characterization of AMELX allelic dropout caused by the failure of AMELX gene amplification in two male cases.

### 3.2. Verification of the Failure of AMELX Gene Amplification in Forensic Samples

**Figure 3:**
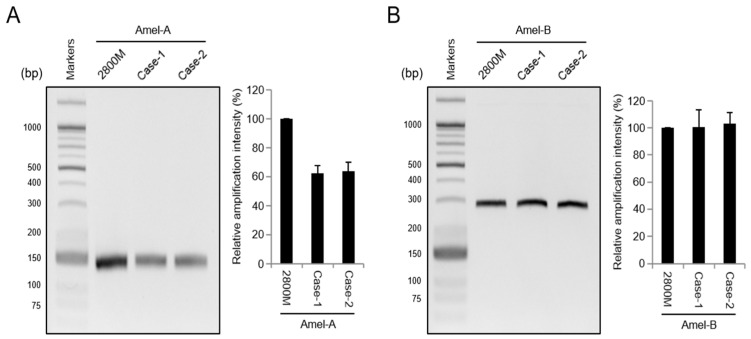
Identification of amelogenin gene amplification in AMELX allelic dropout case samples. The amelogenin gene in AMELX allelic dropout cases (Case-1 and Case-2) was amplified by Amel-A primer (**A**) or Amel-B primer (**B**). Data represent the mean ± standard deviation.

We showed how, in two male cases, the failure of AMELX gene amplification could affect AMELX allelic dropout. In order to examine this carefully, we examined two kinds of previously reported amelogenin primers (Amel-A and Amel-B) to monitor the amplification of the AMELX gene. As the amelogenin primers mentioned above can amplify both AMELX and AMELY genes in a single reaction, the failure of the amelogenin gene (AMELX or AMELY) can be inferred through the intensity of amplification. As shown in Figure 3A, Amel-A displayed a half level of amelogenin gene amplification intensity in two male cases compared to the control DNA. Interestingly, the amplification intensity of the AMELX gene dramatically recovered, similar to that of the control DNA, when amplified with Amel-B, suggesting that the occurrence of an unusual point of mutation in the Amel-A primer-binding region may affect AMELX gene amplification (Figure 3B). As mentioned above, two amelogenin genes can be amplified simultaneously in a single reaction, so the expressed half level of amelogenin gene amplification intensity can be inferred using AMELY gene amplification. As a result, the failure of AMELX gene amplification was judged to be the cause of AMELX allelic dropout in the two commercial kits shown in Figure 2.

**Figure 4:**
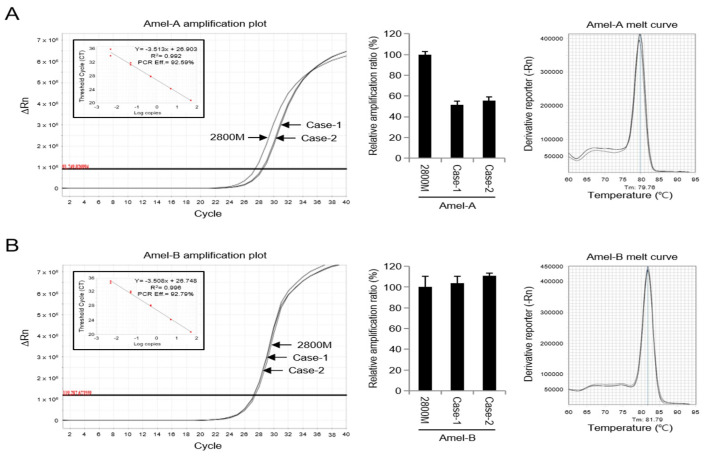
Quantification of amelogenin gene amplification using qPCR in AMELX allelic dropout cases (Case-1 and Case-2). The amplification of the amelogenin gene was quantified via real-time PCR using SYBR^®^ green dye with the Amel-A primer (**A**) or Amel-B primer (**B**). A single melt curve peak indicates that the reaction is specific for the amplification product of the Amel-A primer (Tm: 79.76 °C) or Amel-B primer (Tm: 81.79 °C). Data represent the mean ± standard deviation.

To further determine amelogenin gene amplification in the two male cases, quantitative PCR (qPCR) amplification using both Amel-A and Amel-B was performed, allowing a comparison of the DNA quantity between samples (Figure 4). As shown in Figure 4A, the positive control DNA and two male cases using Amel-A displayed different cycle threshold (Ct) values of 27 and 29, respectively. When Amel-B was used, Ct values were close to 27 and similar to each other (Figure 4B). Additionally, the melt curve of both positive control DNA and two male cases with Amel-B displayed a similar dissociation curve pattern (Figure 4B); however, a slight dissociation curve was observed for Amel-A (Figure 4A). The linear regression lines generated by plotting Ct values against DNA concentration yielded coefficients of correlation (R^2^) ranging from 0.992 to 0.996. These results suggest that the occurrence of unusual point mutations in the Amel-A primer-binding motif in the amelogenin gene may affect AMELX gene amplification and consequently cause AMELX allelic dropout in amelogenin genotyping. 

### 3.3. Validation of Point Mutation of AMELX Locus in Forensic Samples

**Figure 5:**
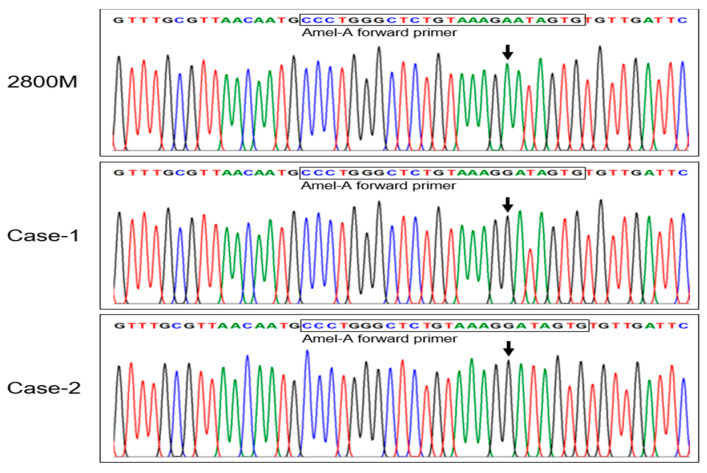
Sequencing of the X chromosome forward strand from positive control DNA (2800 M) and AMELX allelic dropout cases (Case-1 and Case-2). In the AMELX allelic dropout case samples, sequencing analysis of AMELX revealed an A to G transversion in the annealing region of the Amel-A forward primer (arrow). The nucleotide sequence of the Amel-A forward primer is outlined by the rectangle.

To confirm the variant of the AMELX locus for the two male cases, we performed a Sanger sequencing analysis using the Amel-A forward primer and reverse primer (Figure 5). We found a homozygous variant from A→G at base position 7 downstream from the 3′ end in the Amel-A forward primer-binding region; this point mutation was found in both male cases. Based on these results, to verify whether the point mutation identified in both cases also affects AMELX gene amplification, we designed a mutation-specific primer (Amel-A A/G forward) based on sequencing analysis. The newly designed Amel-A A/G forward primer substituted a G base for an A base located at the 7th base position from the 3′ end (Figure 1).

**Figure 6:**
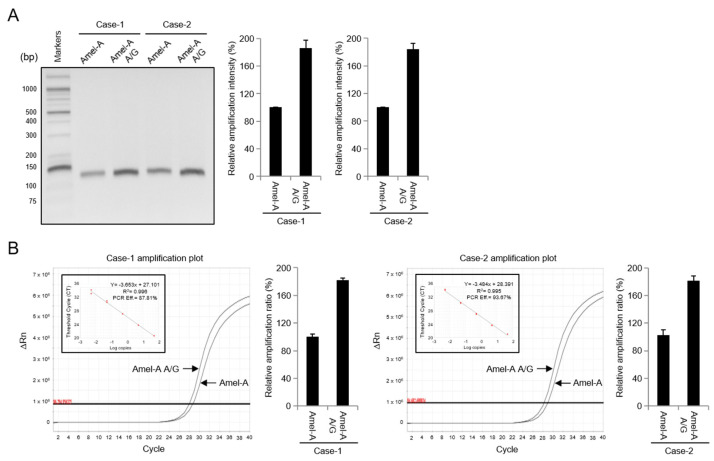
The amplification of amelogenin in AMELX allelic dropout cases (Case-1 and Case-2) was recovered by the Amel-A A/G substituted primer. The amplification of the amelogenin gene was analyzed using the Amel-A primer or Amel-A A/G primer (**A**). The quantification of the amelogenin gene was subjected to real-time PCR using SYBR^®^ green dye with Amel-A primer or Amel-A A/G primer (**B**). Data represent the mean ± standard deviation.

To determine whether the mutation-specific primer (Amel-A A/G forward primer) represents a reliable approach for amelogenin gene amplification, specifically AMELX gene amplification, we amplified the AMELX region. We compared the intensity of amplification between traditional and modified primer pairs for two male cases. As shown in Figure 6A, the level of amplification using mutation-specific primers markedly increased in both cases (Case-1 and Case-2). qPCR amplification with the primer set containing the Amel-A A/G primer and Amel-A primer pair also generated Ct values of 27, 28, and 29 in both cases, respectively (Figure 6B). The linear regression lines generated by plotting Ct values against DNA concentration yielded coefficients of correlation (R^2^) ranging from 0.995 to 0.996.

**Figure 7:**
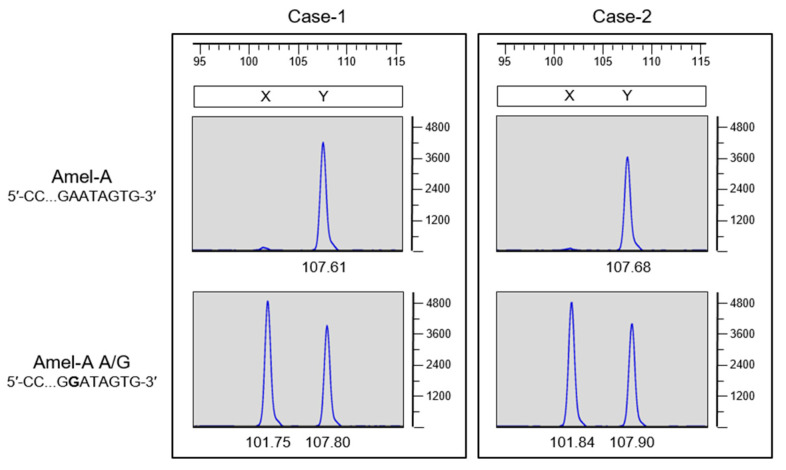
Amelogenin genotyping in AMELX allelic dropout case samples was recovered using the Amel-A A/G-substituted primer. DNA from AMELX allelic dropout cases (Case-1 and Case-2) were subjected to PCR using either the Amel-A forward (FAM-labeled) or Amel-A A/G forward (FAM-labeled) primer. GeneMapper ID-X software was used to size the X (~101 bp) and Y (~107 bp) PCR products. Horizontal axes indicate PCR product sizes in bp, while vertical axes indicate fluorescent intensity in relative units.

To further verify that the Amel-A A/G primer can be used as a reliable method for gene amplification, we determined the allele peak signal resulting from the amplification of the gene in two case samples via capillary electrophoresis STR analysis, and the results are shown in Figure 7. The AMELX allele was not detected in GlobalFiler™ or PowerPlex^®^Fusion kits and was recovered using the Amel-A A/G (FAM-labeled) forward primer. These results imply that the point mutation (A→G) at base position 7 downstream from the 3′ end in the Amel-A forward primer-binding region induced the failure of AMELX gene amplification. Further, the primer set containing the Amel-A A/G forward primer is a suitable option to resolve the failure of AMELX gene amplification in these mutation cases. 

## 4. Discussion

A failure to detect diagnostic targets for sex chromosomes in crime scene samples can increase the risk of errors in gender interpretation. However, alternative identification methods have not been adequately studied and remain an important challenge in forensic diagnosis and applications. In this study, we discovered two male cases of AMELX allelic dropout when analyzing STR genotypes obtained from crime scene evidence. 

To investigate the failure of AMELX gene amplification in forensic samples, we used two established amelogenin primers, Amel-A and Amel-B, which typically amplify both AMELX and AMELY genes together. We found that Amel-A displayed only half the amplification intensity in the two male cases compared to the control DNA, indicating a potential failure of AMELX gene amplification. Furthermore, STR genotyping analysis using two types of commercial kits (Figure 2) confirmed this phenomenon. With the Amel-B primer, the amplification intensity of the AMELX gene dramatically recovered, similar to that of the control DNA. This suggests that the issue with AMELX gene amplification observed with the Amel-A primer could be specific to the Amel-A primer, likely due to an unusual point of mutation in its binding region.

Further, qPCR using both Amel-A and Amel-B primers reinforced this idea. The Ct values obtained when using Amel-A showed a difference between the positive control DNA and the two male cases, suggesting some variability in the amplification efficiency. By contrast, when using Amel-B, the Ct values were similar between the samples. The high coefficients of correlation (R^2^) obtained from plotting Ct values against DNA concentration indicate a strong relationship between DNA quantity and Ct values. Therefore, the observed differences in amplification efficiency between Amel-A and Amel-B suggest that point mutations in the Amel-A primer-binding motif may be responsible for the observed allelic dropout in amelogenin genotyping. These mutations could affect the amplification of the AMELX gene and lead to AMELX allelic dropout in amelogenin genotyping.

Sanger sequencing of the Amel-A forward and reverse primers was then conducted to confirm the AMELX variant in the two male cases. Sequencing revealed a homozygous A→G point mutation in the Amel-A forward primer-binding region of the AMELX gene. This was believed to cause AMELX allelic dropout in genotyping. Therefore, a mutation-specific primer (Amel-A A/G) was designed, which successfully amplified the AMELX gene, making it a reliable solution for cases with this mutation.

Coincidentally, the non-detection of the AMELX gene when using point mutation in this study is consistent with one of several mutation cases (specifically, the point mutation of A→G in Sample 1) reported by Ou et al. in the Chinese population [16]. However, to our knowledge, this point mutation and failure of AMELX gene amplification has not been reported previously in the Korean population. Generally, it is well known that the correct base pair binding for the 3′ terminus of PCR primers is the principle of allele-specific PCR, which forms the basis for the amplification refractory mutation system [18]. However, the mutation identified in this study was discovered in the middle region of the PCR primer, which is rare; therefore, it may be valuable to consider reliable approaches, such as optimizing the primers used for amelogenin genotyping.

In this case study, we suggest that a systematic research approach could help understand AMELX allelic dropout and contribute to a better interpretation of gender identification for forensic samples. In addition, a cohort study of the Korean population is required to understand the variants involved in amelogenin allelic dropout. In this study, while the two male cases provide initial evidence of AMELX allelic dropout due to point mutation, validating these findings with larger Korean population samples could strengthen an understanding of amelogenin allelic dropout. Ultimately, this could help ascertain how widespread the variants involved in amelogenin allelic dropout are within the Korean population and potentially in other populations. And a comparative study involving multiple populations could reveal interesting genetic variations and implications for forensic genotyping.

## 5. Conclusions

In this study, we report two male cases in which the X homology amplification of amelogenin failed due to the same mutation. We systematically investigate and demonstrate that the point mutation (A→G) at base position 7 downstream from the 3′ end in the Amel-A forward primer-binding region led to AMELX allelic dropout cases. To the best of our knowledge, this study is the first report on the X homolog amplification failure of amelogenin caused by a point mutation in the Korean population and provides a reliable approach for AMELX allelic dropout due to rare case mutations. We suggest that a systematic research approach, such as this case study, could help understand AMELX allelic dropout in crime scene samples. In addition, a cohort study of the Korean population is required to further understand the variants involved in amelogenin allelic dropout. An expansion of Korean population studies could help us understand genetic variation within the Korean population and other population groups, enabling a better interpretation of gender identification in forensic genotyping.

## Data Availability

The data presented in this study are available upon request to the corresponding author.
